# Supplementary material for: Rora Regulates Neutrophil Migration and Activation in Zebrafish
Source: Front Immunol. 2022 Mar 4;13:756034. doi: 10.3389/fimmu.2022.756034 (PMC8931656; doi:10.3389/fimmu.2022.756034)
Supplement: Supplementary file 5 [file DataSheet_5.docx]

**
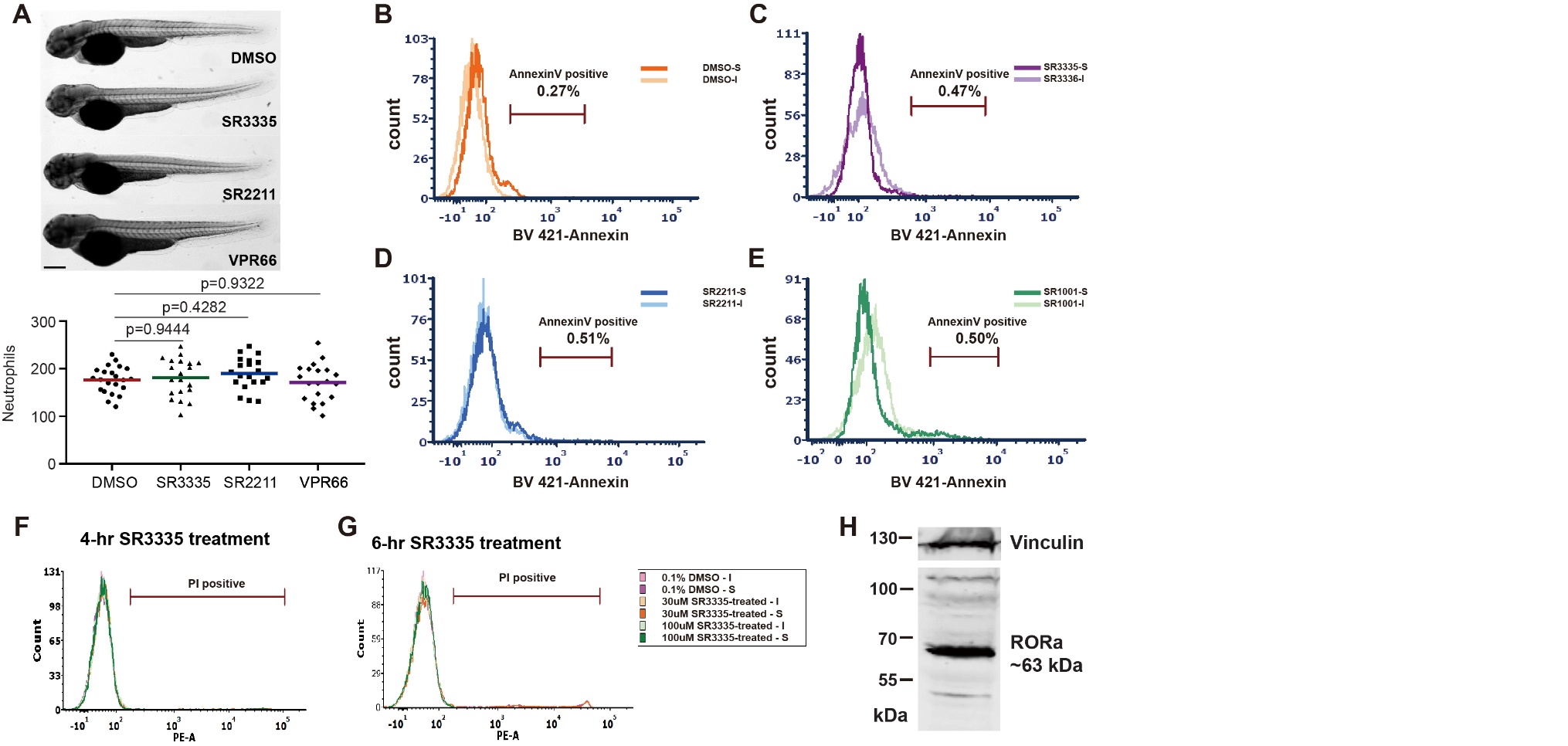
**

**Figure S1. RORα inhibitor treatment does not cause neutrophil death.** (A) Representative images and quantification of total neutrophils in zebrafish larva treated with RORα specific inhibitor (SR3335, 100μM), RORγ specific inhibitor (SR2211, 100μM) or pan-ROR family inhibitor (VPR66, 25μM). *P*-value was calculated with unpaired Student's t-test. Scale bar: 100 µm. (B) Annexin V staining in the total cell population (excluding debris) of primary human neutrophils treated with SR3335 (50μM), SR2211 (50μM), or SR1001 (pan-ROR family inhibitor) (50 µM). Percentage of cells with Annexin V levels above the baseline are shown. S: signal; I: unstained cells. (H) Immunoblot detecting RORα expression in primary human neutrophils. Vinculin is used as a loading control.

**
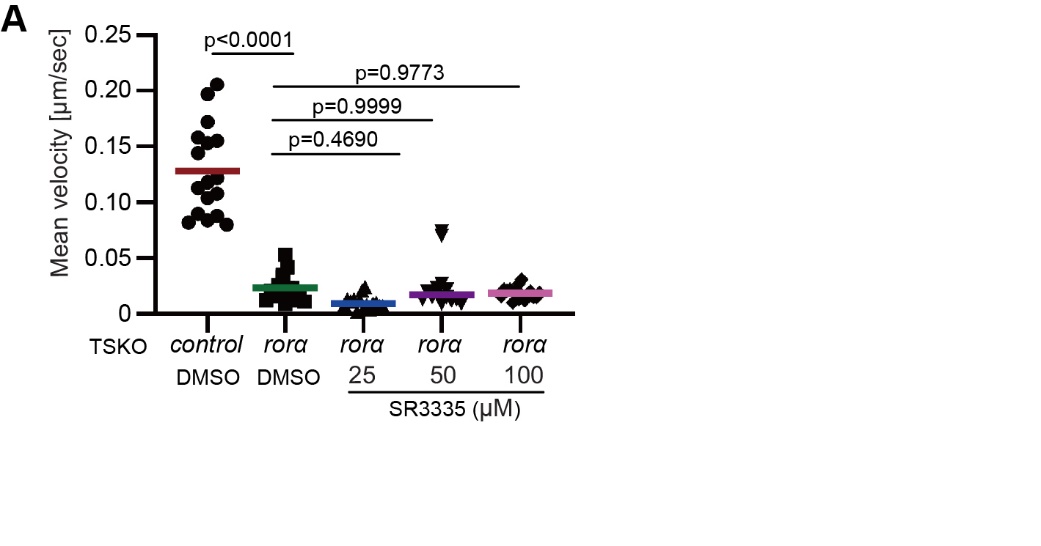
Figure S2. SR3335 treatments does not further decrease neutrophil velocity when *rora* is deleted.** (A) Quantification of neutrophil velocity in zebrafish larva expressing control or *rora* targeting sgRNAs in neutrophils, with additional treatment of DMSO or SR3335 at indicated concentrations, Kruskal–Wallis test.

**Movie S1: Tracked movies of neutrophil motility lines in the head mesenchyme of the vector and the *miR-99*.**

The video shows the motility of neutrophils in 3 dpf vector and miR-99 zebrafish lines. Videos were recorded for 30min with a 1min interval. Representative videos from n = 3 independent experiments with three fish in each group are shown. Scale bar: 100 µm.

**Movie S2: Tracked movies of neutrophil motility in the head mesenchyme treated with vehicle, SR3335, SR2211, or VPR66.**

The video shows the motility of neutrophils in 3 dpf zebrafish larvae treated with DMSO (vehicle control), SR3335, SR2211, or VPR66. Videos were recorded for 30min with a 1min interval. Representative videos from n = 3 independent experiments with three fish in each group are shown. Scale bar: 100 µm.

**Movie S3: SR3335 inhibits chemotaxis of primary human neutrophils.**

Tracks of primary human neutrophils chemotaxis treated with DMSO, SR3335, SR2211, or SR1001 towards fMLP. Videos were recorded for 50min with 30s intervals. Representative videos from n = 4 separate trials are shown. Scale bar: 50 µm.

**Movie S4: Tracked movies of neutrophil motility in the head mesenchyme of the control and Rorα DN lines.**

The video shows the motility of neutrophils in 3 dpf control or Rorα DN zebrafish lines. Videos were recorded for 30 min with 1 min interval. Representative videos from n = 3 independent experiments with three fish in each group are shown. Scale bar: 100 µm.

**Movie S5: Morphology of neutrophils from the control and Rorα DN lines**

The video shows the morphology and stable actin distribution of neutrophils in 3 dpf control or Rorα DN zebrafish lines crossed with a *Tg(mpx:gfp-utrCH)* line. Videos were recorded for 10min with a 1min interval. Representative videos from n = 3 independent experiments with three fish in each group are shown. Scale bar: 20 µm.

**Dataset S1. List of genes that are downregulated in the miR-99 overexpressing neutrophils.**

**Dataset S2. Pathway enrichment analysis of downregulated genes in *miR-99* overexpressing neutrophils.**

**Dataset S3. List of genes that are downregulated in the Roraa-DN overexpressing neutrophils.**

**Dataset S4. Pathway enrichment analysis of downregulated genes in Roraa-DN overexpressing neutrophils.**

**Table 1. Overlap human homologs of genes in the down-regulated DEGs with putative RORα target genes in THP-1 or HUVEC cells.** Genes selected for validation in Figure 7d are highlighted.
